# Supplementary material for: Systematic review and meta-analysis of myopia prevalence in African school children
Source: PLoS One. 2022 Feb 3;17(2):e0263335. doi: 10.1371/journal.pone.0263335 (PMC8812871; doi:10.1371/journal.pone.0263335)
Supplement: S1 Table — (DOCX) [file pone.0263335.s001.docx]

**S1 Table: Quality assessment of full-text articles included in review**

| Study | Is the hypothesis/ aim/ objective of the study clearly described? | Are the main outcomes to be measured clearly described in Introduction or Methods section | Is the source, methods and eligibility criteria for participant selection stated? | Is how the study size was arrived at explained? | Is location and sampling period of study described? | Is a good description of method of measurement given for each variable of interest? | Are the characteristics of the patients included in the study clearly described? | Were the statistical methods well described and analysis well reported? | Are the main findings of the study clearly described? | Is a cautious overall interpretation of results given, considering objectives, results from similar studies? | Total score |
| --- | --- | --- | --- | --- | --- | --- | --- | --- | --- | --- | --- |
| Atowa et al(34) | 1 | 1 | 1 | 1 | 1 | 1 | 1 | 1 | 1 | 1 | 10 |
| Wajuihian & Hansraj (35) | 1 | 1 | 1 | 1 | 1 | 1 | 1 | 1 | 1 | 1 | 10 |
| Chebil et al(36) | 1 | 1 | 1 | 0 | 1 | 1 | 1 | 1 | 1 | 1 | 9 |
| Kedir & Girma(37) | 1 | 1 | 1 | 1 | 1 | 1 | 1 | 1 | 1 | 1 | 10 |
| Soler et al,(38) | 1 | 1 | 1 | 0 | 0 | 1 | 1 | 1 | 1 | 1 | 8 |
| Kumah et al(39) | 1 | 1 | 1 | 1 | 1 | 1 | 1 | 1 | 1 | 1 | 10 |
| Mehari, et al(41) | 1 | 1 | 1 | 0 | 1 | 1 | 1 | 1 | 1 | 1 | 9 |
| Jimenez, et al(42) | 1 | 1 | 1 | 0 | 0 | 1 | 1 | 1 | 1 | 1 | 8 |
| Naidoo, et al(43) | 1 | 1 | 1 | 0 | 1 | 1 | 1 | 1 | 1 | 1 | 9 |
| Yamamah et al(45) | 1 | 1 | 1 | 1 | 1 | 1 | 1 | 1 | 1 | 1 | 10 |
| Nartey et al, (46) | 1 | 1 | 1 | 1 | 1 | 1 | 1 | 1 | 1 | 1 | 10 |
| Anera et al., (47) | 1 | 1 | 1 | 0 | 0 | 0 | 1 | 1 | 1 | 1 | 7 |
| Chukwuemeka (49) | 1 | 1 | 1 | 1 | 1 | 1 | 1 | 1 | 1 | 1 | 10 |
| Alrasheed(50) | 1 | 1 | 1 | 1 | 1 | 1 | 1 | 1 | 1 | 1 | 10 |
| Abdul-Kabir(51) | 1 | 1 | 1 | 1 | 1 | 1 | 1 | 1 | 1 | 1 | 10 |
| Ebri (52) | 1 | 1 | 1 | 1 | 1 | 1 | 1 | 1 | 1 | 1 | 10 |
| Ezinne (53) | 1 | 1 | 1 | 1 | 1 | 1 | 1 | 1 | 1 | 1 | 10 |
| Nakua(54) | 1 | 1 | 1 | 1 | 1 | 1 | 1 | 1 | 1 | 1 | 10 |
| Ndou (55) | 1 | 1 | 1 | 1 | 1 | 1 | 1 | 1 | 1 | 1 | 10 |
| Ahmed, Alrasheed and Alghamdi (56) | 1 | 1 | 1 | 1 | 1 | 1 | 1 | 1 | 1 | 1 | 10 |
| Ovenseri-Ogbomo & Omuemu (57) | 1 | 1 | 1 | 1 | 1 | 1 | 1 | 1 | 1 | 1 | 10 |
| Ovenseri-Ogbomo & Assien (58) | 1 | 1 | 1 | 0 | 1 | 1 | 1 | 1 | 1 | 1 | 9 |
| Assem et al (60) | 1 | 1 | 1 | 1 | 1 | 1 | 1 | 1 | 1 | 1 | 10 |
| Maduka-Okafor et al (61) | 1 | 1 | 1 | 1 | 1 | 1 | 1 | 1 | 1 | 1 | 10 |
