# Supplementary material for: Systematic review and meta-analysis of myopia prevalence in African school children
Source: PLoS One. 2022 Feb 3;17(2):e0263335. doi: 10.1371/journal.pone.0263335 (PMC8812871; doi:10.1371/journal.pone.0263335)
Supplement: S2 File — (DOCX) [file pone.0263335.s003.docx]

**S2. Search terms for Refractive error Africa children prevalence Filters (2000 – 2021)**

(((("refractive errors"[MeSH Terms] OR ("refractive"[All Fields] AND "errors"[All Fields])) OR "refractive errors"[All Fields]) OR ("refractive"[All Fields] AND "error"[All Fields])) OR "refractive error"[All Fields]) AND ((("Africa"[MeSH Terms] OR "Africa"[All Fields]) OR "Africa s"[All Fields]) OR "Africas"[All Fields]) AND (((((("child"[MeSH Terms] OR "child"[All Fields]) OR "children"[All Fields]) OR "child s"[All Fields]) OR "children s"[All Fields]) OR "childrens"[All Fields]) OR "childs"[All Fields]) AND ((((((((("epidemiology"[MeSH Subheading] OR "epidemiology"[All Fields]) OR "prevalence"[All Fields]) OR "prevalence"[MeSH Terms]) OR "prevalance"[All Fields]) OR "prevalences"[All Fields]) OR "prevalence s"[All Fields]) OR "prevalent"[All Fields]) OR "prevalently"[All Fields]) OR "prevalents"[All Fields])

**Translations**

**Refractive error:** "refractive errors"[MeSH Terms] OR ("refractive"[All Fields] AND "errors"[All Fields]) OR "refractive errors"[All Fields] OR ("refractive"[All Fields] AND "error"[All Fields]) OR "refractive error"[All Fields]

**Africa:** "Africa"[MeSH Terms] OR "Africa"[All Fields] OR "Africa's"[All Fields] OR "Africas"[All Fields]

**children:** "child"[MeSH Terms] OR "child"[All Fields] OR "children"[All Fields] OR "child's"[All Fields] OR "children's"[All Fields] OR "childrens"[All Fields] OR "childs"[All Fields]

**prevalence:** "epidemiology"[Subheading] OR "epidemiology"[All Fields] OR "prevalence"[All Fields] OR "prevalence"[MeSH Terms] OR "prevalance"[All Fields] OR "prevalences"[All Fields] OR "prevalence's"[All Fields] OR "prevalent"[All Fields] OR "prevalently"[All Fields] OR "prevalents"[All Fields]
