# Supplementary material for: Systematic review and meta-analysis of myopia prevalence in African school children
Source: PLoS One. 2022 Feb 3;17(2):e0263335. doi: 10.1371/journal.pone.0263335 (PMC8812871; doi:10.1371/journal.pone.0263335)
Supplement: S5 File — (DOCX) [file pone.0263335.s006.docx]

S5: Funnel plots and 95% confidence intervals of Myopia by age in categories
