# Supplementary material for: Systematic review and meta-analysis of myopia prevalence in African school children
Source: PLoS One. 2022 Feb 3;17(2):e0263335. doi: 10.1371/journal.pone.0263335 (PMC8812871; doi:10.1371/journal.pone.0263335)
Supplement: S7 File — The vertical axis is the log proportion of Myopia, and the horizontal axis represents year of publication. Each dark dot represented one selected study, and the size of each dark dots corresponds to the weight assigned to each study. Given the slope of the regression line has descending slightly in this figure, this could be interpreted as publication of year increased, the proportion of myopia decreased and, this relationship did not differ statistically (p = 0.5512). (DOCX) [file pone.0263335.s008.docx]

S7. A meta-regression analysis of Myopia by year of publication. The vertical axis is the log proportion of Myopia, and the horizontal axis represents year of publication. Each dark dot represented one selected study, and the size of each dark dots corresponds to the weight assigned to each study. Given the slope of the regression line has descending slightly in this figure, this could be interpreted as publication of year increased, the proportion of myopia decreased and, this relationship did not differ statistically (p= 0.5512)
